# A Comparative Study of Feature Selection Methods for the Discriminative Analysis of Temporal Lobe Epilepsy

**DOI:** 10.3389/fneur.2017.00633

**Published:** 2017-12-06

**Authors:** Chunren Lai, Shengwen Guo, Lina Cheng, Wensheng Wang

**Affiliations:** ^1^Department of Biomedical Engineering, South China University of Technology, Guangzhou, China; ^2^Department of Radiation Oncology, The People’s Hospital of Gaozhou, Gaozhou, China; ^3^Medical Imaging Center, Guangdong 999 Brain Hospital, Guangzhou, China

**Keywords:** temporal lobe epilepsy, magnetic resonance images, cortical features, feature selection, classification

## Abstract

It is crucial to differentiate patients with temporal lobe epilepsy (TLE) from the healthy population and determine abnormal brain regions in TLE. The cortical features and changes can reveal the unique anatomical patterns of brain regions from structural magnetic resonance (MR) images. In this study, structural MR images from 41 patients with left TLE, 34 patients with right TLE, and 58 normal controls (NC) were acquired, and four kinds of cortical measures, namely cortical thickness, cortical surface area, gray matter volume (GMV), and mean curvature, were explored for discriminative analysis. Three feature selection methods including the independent sample *t*-test filtering, the sparse-constrained dimensionality reduction model (SCDRM), and the support vector machine-recursive feature elimination (SVM-RFE) were investigated to extract dominant features among the compared groups for classification using the support vector machine (SVM) classifier. The results showed that the SVM-RFE achieved the highest performance (most classifications with more than 84% accuracy), followed by the SCDRM, and the *t*-test. Especially, the surface area and GMV exhibited prominent discriminative ability, and the performance of the SVM was improved significantly when the four cortical measures were combined. Additionally, the dominant regions with higher classification weights were mainly located in the temporal and the frontal lobe, including the entorhinal cortex, rostral middle frontal, parahippocampal cortex, superior frontal, insula, and cuneus. This study concluded that the cortical features provided effective information for the recognition of abnormal anatomical patterns and the proposed methods had the potential to improve the clinical diagnosis of TLE.

## Introduction

Epilepsy, affecting approximately 50 million patients worldwide, has attracted increasing attention of many investigators. Among various epilepsy categories, temporal lobe epilepsy (TLE) is the most common drug-resistant category (1), which originates in the temporal lobe and involves structural and functional abnormalities of the brain (2–4). For patients with epilepsy for whom treatment with medicines is not effective, surgical treatment is usually considered as an eventual option. Thus, it is very important to determine effective and objective biomarkers to differentiate early TLE patients from normal controls (NC), so as to control seizures and prevent deteriorating.

During the past two decades, abundant voxel-based morphometry (VBM) studies have shown that structural changes in patients with TLE extend beyond the mesial temporal structures. There were differences in the extent of anatomical damage between hemispheres (5, 6) and the morphological abnormalities were more widespread in the left temporal lobe epilepsy (LTLE) with gray matter volume (GMV) loss, especially in the hippocampus, the parahippocampal gyrus, and the entorhinal cortex (7). Some resting-state fMRI studies suggested that significant decreases of the regional homogeneity (ReHo) were observed mainly in the default mode network (DMN), including the precuneus, the posterior cingulate gyrus, the bilateral inferior lateral parietal, and the mesial prefrontal cortex (8, 9). Similar changes were detected in the low-frequency fluctuation (ALFF) (10, 11). Functional magnetic resonance imaging (MRI) findings have indicated that patients with LTLE have significantly lower scores in verbal fluency than controls while compared to the right TLE (RTLE) (12). At the individual level, more patients with LTLE showed decreased or disrupted functional connectivity while a reverse pattern was found in RTLE (13). Neuropathological research has revealed various patterns of neuronal cell loss within the hippocampus and adjacent temporal lobe structures in the brain of the TLE patients (14). High-resolution MRI illustrated that the focal cortical dysplasia was a cause of medically intractable partial epilepsy (15). Morphological features of the cerebral cortex such as cortical thickness (CTh), surface area, GMV, and mean curvature (MCu) have been found to be associated with pathogenesis of either the LTLE or the RTLE. Previous studies indicated that there existed asymmetric reduction of cortical surface area (CSA) in the ipsilateral mesial and the anterior temporal lobe subregions (16), MCu abnormality in the bilateral insula (17), overall cortical thinning (18), and gray volume loss (19).

Based on the morphological and functional features of the brain, machine learning techniques such as the support vector machine (SVM), artificial neural network, clustering, and Bayesian networks, have been widely used in distinguishing patients with brain disorders from healthy controls (20–24). Existing studies have demonstrated that neuroimaging data were considered potential biomarkers for LTLE and RTLE diagnosis (25–30). For example, anatomical connectivity was reported for the separation of patients with LTLE from normal individuals with accuracy up to 93 and 90.0% for the RTLE (27), voxel-based MRI classification using diffusion tensor imaging (DTI), and T1-weighted MRI of the LTLE or RTLE patients could reach 90–100% (28, 29), the large-scale functional brain network measures as informative biomarkers for epilepsy pattern classification achieved a cross-validated classification accuracy of 83.9% (31). When a linear classifier was used to discriminate the LTLE from RTLE, hippocampal asymmetry could obtain 94% classification accuracy. More recently, the cortical measures were applied to automatically classify epilepsy patients with mesial temporal sclerosis (32).

In machine learning, feature selection is a vital step for extracting meaningful and relevant features to build an efficient classification model, reduce computation complexity, and boost its generalization ability (33–35). Recently, feature selection exhibited great advantages on biomedical studies like genomic (36) and proteomic studies (37), which usually had extremely large feature dimensionality and a small number of samples. In general, the feature selection methods can be divided into three categories according to the interaction with the estimation of the classification model: (1) the filter methods (38), the selection process is independent of classifiers and rank features according to the intrinsic properties, (2) the wrapper methods, which utilize the model’s predictive power to rank subsets of features (39), and (3) the embedded methods, where feature selection interacts with the machine learning process. The filter methods including the *F*-statistic, *t*-test, and principle component analysis, have the benefit of low computational cost, while the wrapper methods are more superior compared to the filter methods by taking discriminative power into consideration. Due to considering interaction among features, embedded methods such as the correlation-based feature selection (40) and support vector machine-recursive feature elimination (SVM-RFE) (41) showed excellent performance in pattern recognition research (32).

Although feature selection methods have been applied in some of the studies on the TLE classification from the neuroimaging data, it still remains uncertain to systematically explore effective feature selection strategies and perform discriminate analysis of cortical surface features from MRI among the LTLE and RTLE patients and the NC. In this study, we investigated feature selection methods to distinguish patients with LTLE and RTLE from the NC. Four morphological measures including the CTh, the CSA, the GMV, and the MCu, were explored for discriminative analysis. Three feature selection methods—the *t*-test filtering, the sparse-constrained dimensionality reduction model (SCDRM), and the SVM-RFE were compared and analyzed. Furthermore, the dominant cortical features and the corresponding brain regions with significant discriminative ability among compared groups were discussed.

## Materials and Methods

### Subjects and Data Acquisition

Forty-one LTLE patients, 34 RTLE patients, and 58 demographically matched NCs were enrolled in this study. Table 1 shows the demographic and clinical information of participants. The patients with seizure onset were determined by experienced physicians. All participant subjects were right-handed native Chinese cohorts recruited from Guangdong 999 hospital, and this study was approved by the Research Ethics Review Board of Guangdong 999 hospital. Written informed consent was obtained in accordance with the Helsinki Declaration prior to their inclusion. The inclusion criteria were as follows: (1) The seizure types and epileptic syndromes were diagnosed according to the classification of the International League Against Epilepsy and determined by the comprehensive evaluation standards including detailed history, neuropathological examination, electroencephalography (EEG) recordings, and MRI inspection; (2) continuous interictal-ictal scalp video EEG showed interictal epileptiform discharges of unilateral temporal origin.

**Table 1:** Demographic and clinical information of participants.

Cohorts	Sample size	1.5T/3.0T	Gender (M/F)	Age (years)	Education (years)	Duration of episode (years)	Onset of epilepsy (years)
Left TLE	41	21/20	23/18	25.5 ± 8.2	9.4 ± 2.7	17.4 ± 9.3	9.4 ± 6.5
Right TLE	34	18/16	18/16	25.0 ± 8.5	8.2 ± 1.7	16.3 ± 9.0	9.3 ± 7.9
Normal controls	58	28/30	29/29	23.2 ± 4.1	12.9 ± 4.0	–	–

All volumetric MRI were acquired with a 1.5T (*n* = 67) or 3.0T (*n* = 66) MRI scanner (1.5T Philips Intera; 3.0T GE Signa HDxT) using the T1-weighted three-dimension magnetization prepared rapid gradient echo (3D-MPRAGE) sequence according to following parameters: repetition time (TR) = 25 ms/8.84 ms, echo time (TE) = 4.6 ms/3.51 ms, matrix size = 256 × 256, voxel dimensions = 0.94 mm × 0.94 mm × 1.2 mm, flip angle = 30°, field of view (FOV) = 240 mm × 240 mm, and slice thickness = 1.2 mm. Approximately 140 isotropic images with voxel size of 1 mm × 1 mm × 1 mm were acquired.

### Feature Extraction

3D T1-weighted DICOM images were converted to NIfTI (neuroimaging informatics technology initiative) format and their orientation was checked using the software MRIcron (http://people.cas.sc.edu/rorden). Cortical morphological measures were computed automatically using the FreeSurfer image analysis software (freely available at http://surfer.nmr.mgh.harvard.edu/). The detailed procedures are described elsewhere (42–45). Briefly, this processing included motion correction and averaging of multiple volumetric T1-weighted images, removal of non-brain tissue using a hybrid watershed and surface deformation procedure, automated Talairach transformation, segmentation of the subcortical white matter and deep gray matter, volumetric normalization of the structure intensity, tessellation of the gray matter and white matter boundaries, automated topology correction, and surface deformation following intensity gradients to optimally place the gray/white and gray/cerebrospinal fluid (CSF) borders at the location where the greatest shift in intensity defined the transition to the other tissue class. Once the cortical models are completed, several deformable procedures could be performed for further data processing and analysis including surface inflation, registration to a spherical atlas, which is based on individual cortical folding patterns to match cortical geometry across subjects, and parcelation of the cerebral cortex into units with respect to gyral and sulcal structure. This method uses both intensity and continuity information from the entire three-dimensional MRI volume in segmentation and deformation procedures to produce representations of the CTh and to calculate the closest distance from the gray/white boundary to the gray/CSF boundary at each vertex on the tessellated surface. After these steps, a mesh model of the cortical surface was generated and the measures were calculated according to the Desikan–Killiany atlas, which divided the cortical surface into 34 distinct cortical regions of interest in each hemisphere. Therefore, 68 features of each type of measure such as the CTh, the surface area, the GMV, and the MCu were obtained.

### Feature Selection

The purpose of feature selection is to optimize the number of features in the subsequent machine learning model to enhance performance and generalizability. The original data usually contain redundant features, which provide no more information than currently selected features, and irrelevant features, which provide no useful information in any context; therefore, these features should be removed. Moreover, feature selection is also useful as a part of the data analysis process, which could determine the importance of each feature in classification or prediction, revealing the relationship between the features.

The univariate *t*-test method, as one of the most commonly used approaches was explored. A two-sample *t*-test was performed to compute the statistical significance value, and then the features with significant difference between the compared groups were extracted.

(1) $$T=\frac{\bar{X_{1}}-\bar{X_{\text{2}}}}{\sqrt{\frac{\text{(}n_{\text{1}}-1\text{)}S_{\text{1}}{\;}^{\text{2}}+\text{(}n_{\text{2}}-1\text{)}S_{\text{2}}{\;}^{\text{2}}}{n_{\text{1}}+n_{\text{2}}-\text{2}}\left(\frac{\text{1}}{n_{\text{1}}}+\frac{\text{1}}{n_{\text{2}}}\right)}}$$

Where *n*_1_ and *n*_2_ denote the sample sizes, ${\bar{X}}_{1}$ and ${\bar{X}}_{2}$ denote the means, while $S_{1}{\;}^{2}$ and $S_{2}{\;}^{2}$ denote the SDs.

The sparsity-constrained dimensionality reduction was based on the least squares problems model, which was introduced a regularization term on model parameters to enforce sparsity using the *l*_2–1_-norm (46).

(2) $$\underset{W}{\text{min}}\sum_{i=1}^{n}{∥W^{T}x_{i}\;-\;y_{i}{∥}_{2,1}^{\;}}_{\;}\;+\;{\lambda R(W)}_{\;}$$

where $\left\{x_{1},\;x_{2},\cdots ,x_{n}\right\}\in R^{d}$ was the training set and $\left\{y_{1},y_{2},\cdots ,y_{n}\right\}\in R^{c}$ denoted the corresponding class labels, **W** was the parameter matrix of size *d* × *c*, and ‖.‖ represented the Frobenius norm of the matrix and λ∈[0,1] was a constant coefficient.

R(**W**) was defined as

(3) $$R(W)=\sum_{i=1}^{d}\|w^{i}{\|}_{2,1}$$

Therefore, the model with the regularization term in Eq. 4 is given by

(4) minWJ(W)=∑i=1n∥WTxi−yi∥ 2,1   + λR(W) =∥XTW−Y∥2,1 + λ∥W∥2,1

The last term in Eq. 4 was a penalty or regularization term on the model parameters to enforce sparsity. An effective iterative algorithm could be used to solve the optimization problem in the model, and the coefficients in the parameters matrix represented the weights of the corresponding features.

The SVM methods were based on recursive feature elimination (SVM-RFE) (41), which eliminated features that were inessential or uninformative for discrimination, and retained the most discriminative features. A feature-ranking strategy based on correlation coefficients was used in the learning model, whereby each feature was given a weight, which represented its magnitude of separation; therefore, the features with the highest weights were the most informative.

### SVM Classification

The SVM classifier, a supervised machine learning algorithm, was used to perform the classification. The LIBSVM toolbox was downloaded from https://www.csie.ntu.edu.tw/~cjlin/libsvm/. To verify the classifier, a nested cross-validation (CV) was applied in this study. In the inner CV loop, a fivefold cross validation and a grid search algorithm was leveraged to determine the optimal parameters for each classifier. During the outer CV loop, the leave-one-out cross validation strategy was employed to train and test each classifier. Three performance measures (accuracy, sensitivity, and specificity) were used to evaluate its performance, which was given according to the following equations:

(5) $$\text{Accuracy}=\frac{\text{TP}+\text{TN}}{\text{TP}+\text{FN}+\text{TN}+\text{FP}}$$

(6) $$\text{Sensitivity}=\frac{\text{TP}}{\text{TP}+\text{FN}}$$

(7) $$\text{Specificity}=\frac{\text{TN}}{\text{TN}+\text{FP}}$$

where TP was the proportion of true positives, which denotes the number of patients correctly predicted; TN, was the proportion of true negatives, which corresponds to the number of subjects correctly classified as the NC; FN, namely, the false negatives, represented the number of individuals incorrectly identified as NC; and the false positives (FP) was the number of the healthy individuals incorrectly identified as patients.

To obtain the best performance and explore dominant features associated with core brain regions, the retained features extracted by the feature selection methods were sorted by the *p-*value from the *t*-test, coefficients in the SCDRM, and weights in the SVM-RFE, which represented the importance of each feature, and then the SVM added the feature one by one in order to evaluate their performance. Therefore, the features with the best performance were determined. To further evaluate the performance of the classifier using the extracted essential features, the corresponding receiver operating characteristic (ROC) curve was created and then the area under the curve (AUC) was computed.

## Results

### Mean Cortical Surface Feature Maps

**Figure 1:**
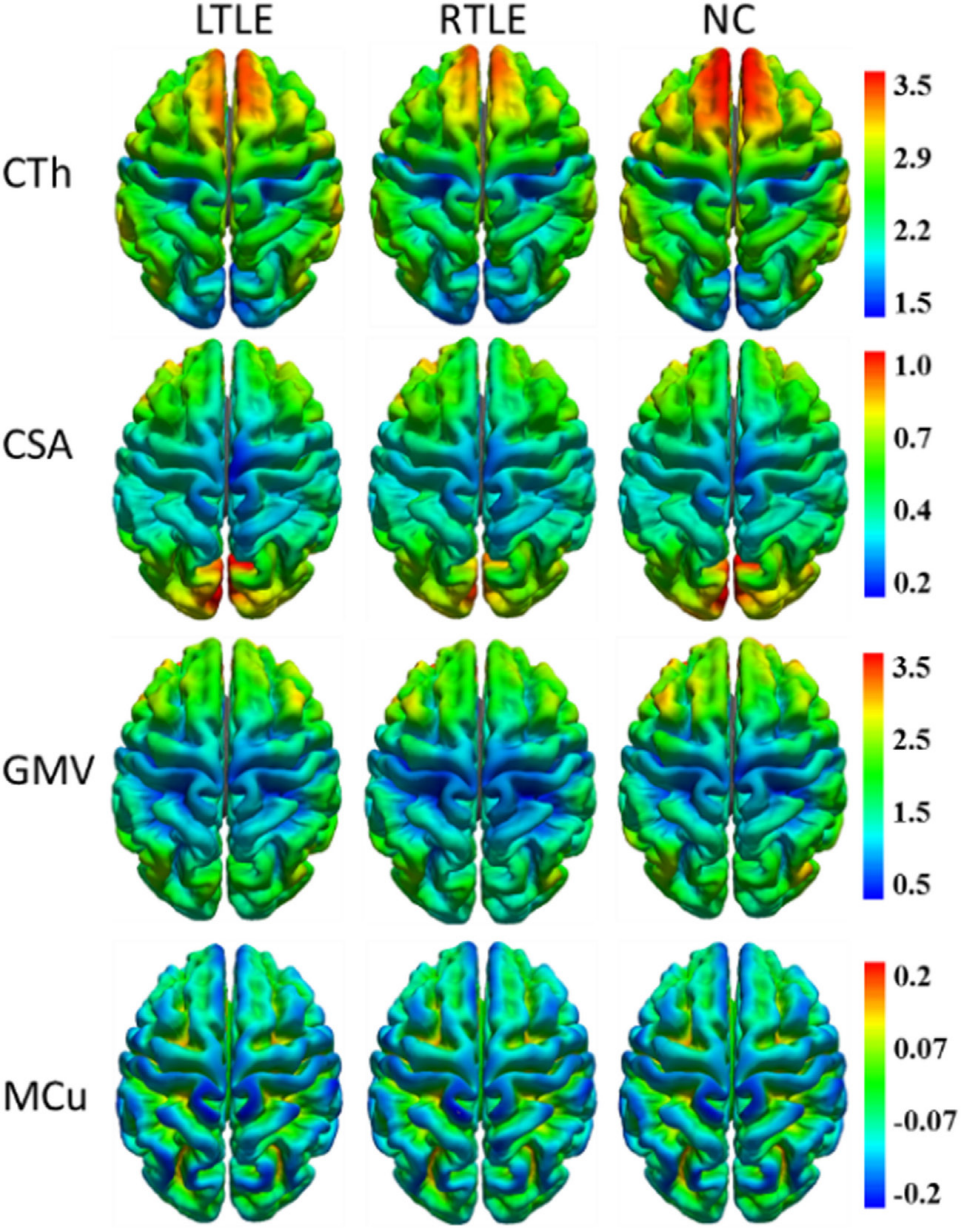
Mean cortical surface feature maps in cortical thickness (CTh), cortical surface area (CSA), gray matter volume (GMV), and mean curvature (MCu) of the left TLE (LTLE), right TLE (RTLE) and normal controls (NC), respectively. Colors from blue to red denote the feature increase from the lowest value to the highest value.

To visualize four types of cortical surface features, the mean maps of the three compared groups were calculated and are shown in Figure 1.

### Visual Inspection

During the diagnostic workup of epilepsy, the patients with seizure onset were comprehensively determined by clinical symptoms, neurobehavioral tests, EEG recordings, and MRI inspection. Despite enormous improvements in MRI scanners and sequences, MRI inspection in the TLE diagnosis was often reported to be unremarkable. In this study, there were 24.4% of LTLE and 23.5% of RTLE participants who did not exhibit distinctive signs in MRI visual analysis. More specifically, 10 of 41 LTLE and 8 of 34 RTLE patients were not identified in magnetic resonance (MR) scans. Besides, ipsilateral hippocampal sclerosis, focal cortical dysplasia, or both of them were visually identified in the other patients, which was one of the primary approaches for determining the lateralization of TLE.

### Statistical Analysis

**Figure 2:**
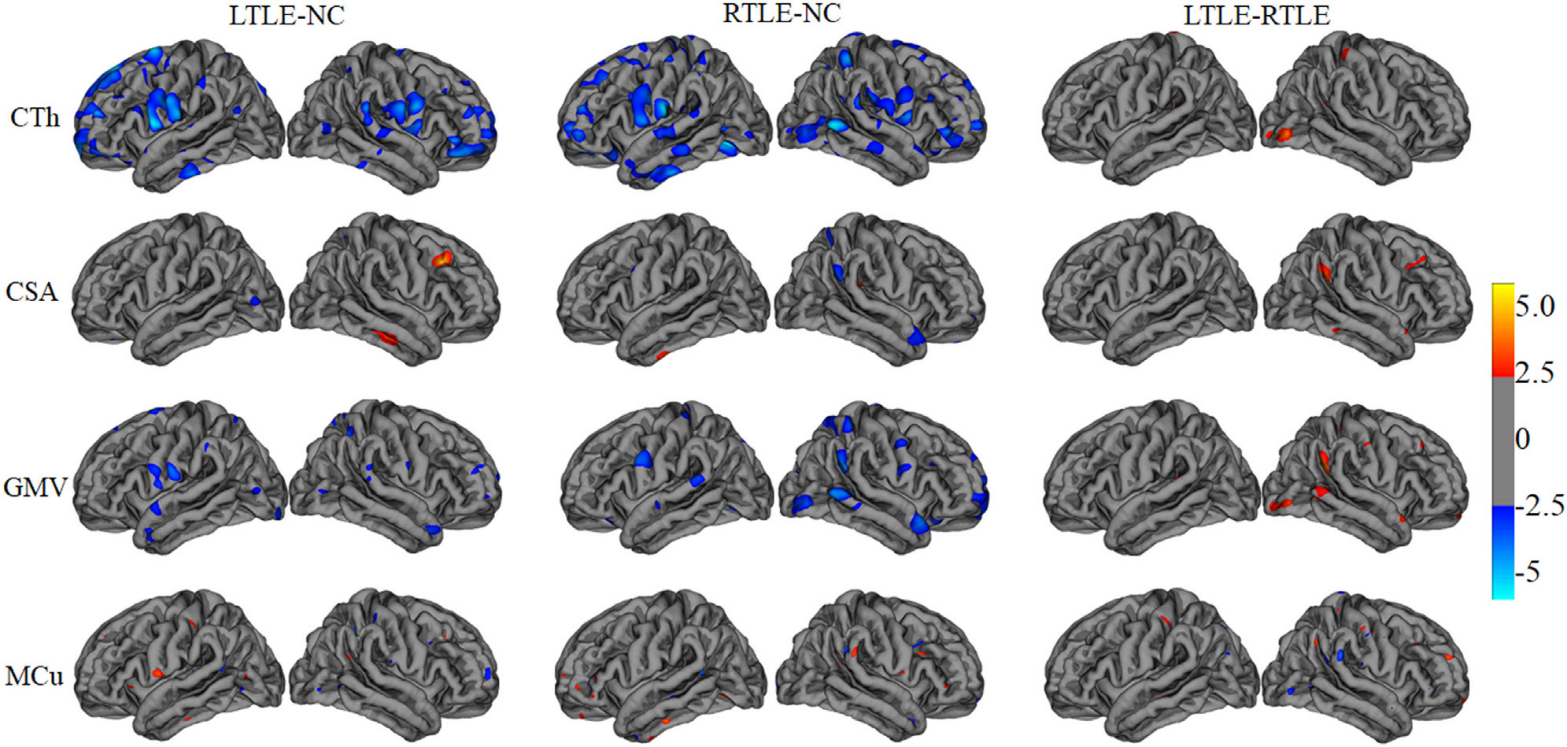
The statistical differences among left TLE (LTLE), right TLE (RTLE), and normal controls (NC) on cortical thickness (CTh), cortical surface area (CSA), gray matter volume (GMV), and mean curvature (MCu). The top line shows that the CTh in brain regions (listed in Table 2 and marked with blue color) reduced significantly in LTLE, RTLE group compared with NC group, while CTh value of a few of brain regions with red color increased significantly in LTLE group compared with RTLE group. Similarly, the second, third, and last lines represent the significant differences of CSA, GMV, and MCu in compared groups.

A tool within FreeSurfer called QDEC was applied to identify structural difference among LTLE, RTLE patients, and NC subjects on CTh, surface area, GMV, and MCu. Age and sex were used as nuisance factor and the statistical *p*-value was set at *p* < 0.01. Finally, the cortical feature differences between two groups patients were mapped onto a standard brain template called Desikan–Killiany atlas and displayed in Figure 2.

**Figure 3:**
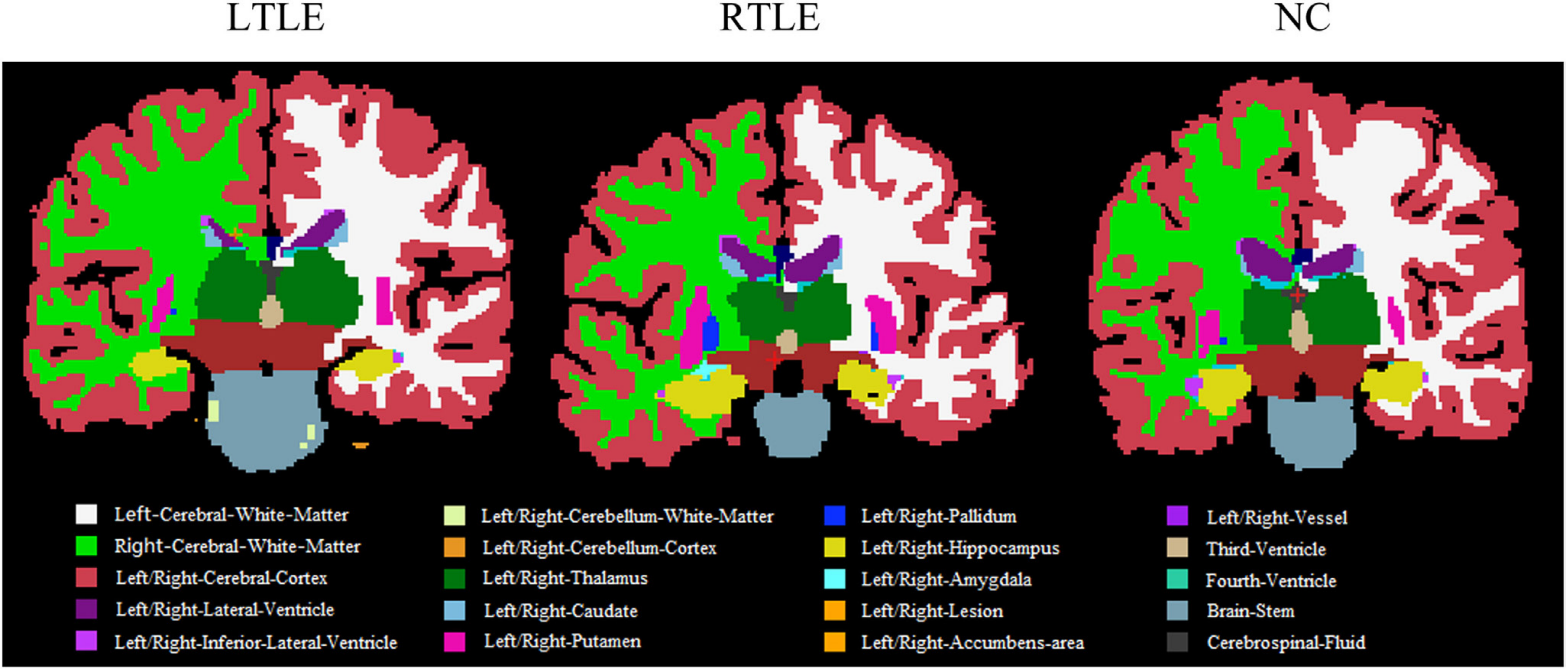
Sectional images of subcortical structures in left TLE (LTLE), right TLE (RTLE), and normal controls (NC) subjects.

Furthermore, to investigate differences or changes of subcortical structures of brain among compared groups, we applied an automatic subcortical segmentation pipeline within FreeSurfer to assign each voxel of normalized brain volume into 40 labels (47). Therefore, the voxel number (volume) of 40 brain regions was obtained and then two sample *t*-test method was used to explore the statistical significance of the 40 features among LTLE-NC and RTLE-NC comparison. As listed in Table 2, the left hippocampus, left thalamus, CSF, posterior cerebral cortex, central cerebral cortex, left ventral diencephalon, and left ventricle changed significantly in the LTLE, while the RTLE patients were found abnormal in the right hippocampus, right thalamus, left thalamus, left cerebellum cortex, right lateral ventricle, right cerebellum cortex, third ventricle, right accumbens area, and left amygdala. Figure 3 shows three sectional images based on the atlas with 40 labeling regions of subcortical structures in LTLE, RTLE, and NC groups, respectively.

**Table 2:** Brain regions with significant differences in subcortical structure of compared groups.

Left TLE (LTLE)-normal controls (NC)		Right TLE (RTLE)-NC		LTLE-RTLE	
Brain region	*p*-Value	Brain region	*p*-Value	Brain region	*p*-Value
Left hippocampus	<0.001	Right hippocampus	<0.001	Right hippocampus	<0.001
Left thalamus	<0.001	Right thalamus	0.002	Left hippocampus	0.0002
Cerebrospinal fluid	0.002	Left thalamus	0.011	Right thalamus	0.04
Posterior cerebral cortex	0.011	Left cerebellum cortex	0.012	Right ventral diencephalon	0.05
Central cerebral cortex	0.013	Right lateral ventricle	0.015		
Left ventral diencephalon	0.017	Right cerebellum cortex	0.015		
Left lateral ventricle	0.02	Third ventricle	0.02		
		Right accumbens area	0.035		
		Left amygdale	0.05		

### Classification Performance

The results of various experiments using the three feature selection strategies and four cortical measures to the classification scenarios are shown in Table 3.

**Table 3:** Classification performance.

Group	Features	Total feature number	SVM-*t*-test				SVM-SCDRM				SVM-RFE			
			Optimal feature number	ACC (%)	SEN (%)	SPE (%)	Optimal feature number	ACC (%)	SEN (%)	SPE (%)	Optimal feature number	ACC (%)	SEN (%)	SPE (%)
LTLE-NC	CTh	68	11	80.81	86.21	73.17	49	76.77	86.21	63.41	22	85.86	82.93	87.93
	CSA	68	6	73.74	77.59	68.29	17	73.74	86.21	56.10	18	86.87	78.05	93.10
	GMV	68	26	80.81	86.21	73.17	29	79.80	87.93	68.29	21	86.87	78.05	93.10
	MCu	68	19	73.74	84.48	58.84	28	80.81	87.93	70.73	45	86.87	80.49	91.38
	CTh + CSA + GMV + MCu	272	35	80.81	87.93	70.73	51	86.87	87.93	85.37	45	94.95	87.80	100

RTLE-NC	CTh	68	3	76.09	84.48	61.76	18	80.43	91.38	61.76	17	84.78	64.71	96.55
	CSA	68	12	81.52	86.21	73.53	19	80.43	91.38	61.76	21	84.78	67.65	94.83
	GMV	68	26	80.43	94.83	55.88	22	80.43	95.66	52.94	18	84.78	64.71	96.55
	MCu	68	5	69.57	89.66	35.29	29	80.43	84.48	73.53	26	85.87	73.53	93.10
	CTh + CSA + GMV + MCu	272	54	83.70	89.66	73.53	45	85.87	91.38	76.47	58	96.76	94.12	98.28

LTLE-RTLE	CTh	68	15	72.0	85.82	82.93	33	69.33	55.88	80.49	20	85.33	90.24	79.41
	CSA	68	12	82.67	76.47	87.80	23	84.0	79.41	87.80	8	92.00	97.56	85.29
	GMV	68	21	78.67	67.65	87.80	46	82.67	82.35	82.93	17	89.33	95.12	82.35
	MCu	68	12	69.33	47.06	87.80	14	80.0	73.53	85.37	25	81.33	85.37	76.47
	CTh + CSA + GMV + MCu	272	20	89.33	88.24	90.24	46	84.0	76.47	90.24	47	96.00	95.12	97.06

*ACC, accuracy; SEN, sensitivity; SPE, specificity; CTh, cortical thickness; CSA, cortical surface area; GMV, gray matter volume; MCu, mean curvature; SVM, support vector machine; SCDRM, sparse-constrained dimensionality reduction model; SVM-RFE, support vector machine-recursive feature elimination; LTLE, left TLE; NC, normal control; RTLE, right TLE*.

From Table 3, we can observe that the SVM-REF achieved the highest performance (most classifications with more than 84% accuracy), followed by the SCDRM, and the *t*-test. It can be seen that the surface area and GMV exhibited the prominent discriminative ability. Moreover, when four types of measure were combined as input features of the SVM-RFE, the highest classification accuracy could reach up to 96.76%, and the optimal feature numbers with the highest performance of the LTLE-NC, RTLE-NC, and LTLE-RTLE classification were 45, 58, and 47, respectively.

In *t*-test feature selection process, the SVM classifier obtained the highest accuracy from 70 to 83% when applying a single measure among the LTLE-NC, RTLE-NC, LTLE-RTLE comparisons. While all of four measures were combined, the classification accuracy was increased to 80.71, 83.70, and 89.33%, respectively. Among the four measures, the CSA exhibited better discriminative ability than the other three measures.

With regard to the SCDRM feature selection method, the highest classification accuracies of the SVM classifier using the single measure were 70–84% among the LTLE-NC, RTLE-NC, and LTLE-RTLE comparisons. The performances of the SVM classifier were significantly improved with the combination of the four measures, such that the accuracies were increased to 86.87, 85.87, and 84.0% in LTLE-NC, RTLE-NC, and LTLE-RTLE comparisons.

**Figure 4:**
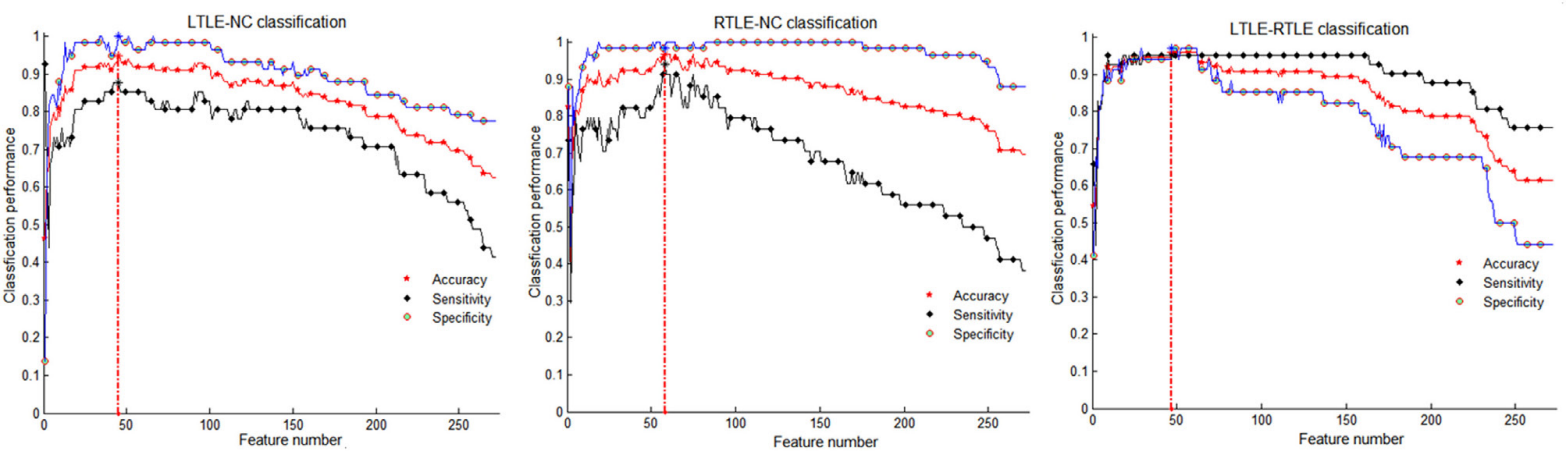
Classification performance among left TLE (LTLE)-normal controls (NC), right TLE (RTLE)-NC, and LTLE-RTLE comparisons using support vector machine-recursive feature elimination strategy by combining all four measures. Red dash lines present optimal classification feature number. Accuracy, sensitivity, and specificity fluctuation during feature selection process show in red, black, and blue straight line, respectively.

**Figure 5:**
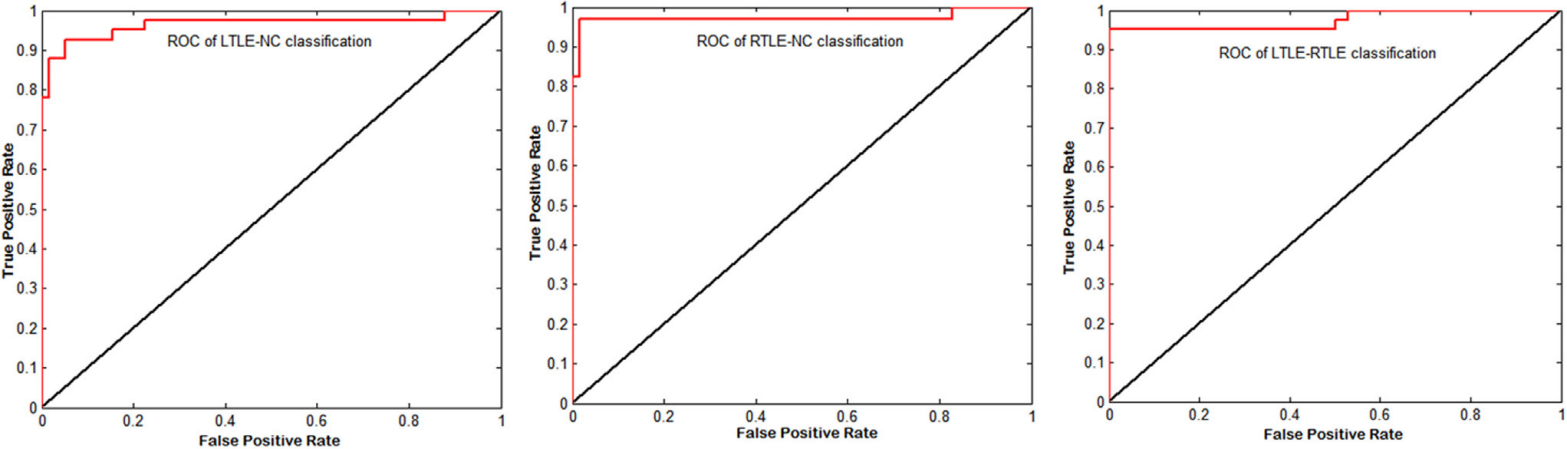
Corresponding receiver operating characteristic (ROC) curves of left TLE (LTLE)-normal controls (NC), right TLE (RTLE)-NC, and LTLE-RTLE comparisons using support vector machine-recursive feature elimination by combining all four measures. The areas under the curves were 0.9651, 0.9731, and 0.9749, respectively.

Table 3 shows that the SVM-RFE achieved much better performance than the other two feature selection methods. The highest accuracies were from 81.3 to 92.0% in the single measure classification. As shown in Tables 3 and 4 and Figures 4 and 5, after the four measures were combined, the SVM classifier obtained much better performance such as the highest accuracies of 94.95% using 45 features in LTLE-NC, 96.76% using 58 features in RTLE-NC, and 96.0% using 47 features in LTLE-RTLE, respectively. The changes in classification performance with the increase in combined measures are shown in Figure 4. The ROC curves are shown in Figure 5.

**Table 4:** The top 15 discriminative features for left TLE (LTLE)-normal controls (NC), right TLE (RTLE)-NC, and LTLE-RTLE classification selected by the support vector machine-recursive feature elimination classifier using combined measures, including cortical thickness (CTh), cortical surface area (CSA), gray matter volume (GMV), and mean curvature (MCu).

Ranking	LTLE-NC	RTLE-NC	LTLE-RTLE
1	Left entorhinal (CSA)	Right rostral middle frontal (CSA)	Right cuneus (CSA)

2	Left insula (CTh)	Right superior parietal (MCu)	Right superior frontal (CSA)

3	Left rostral middle frontal (CTh)	Left insula (CTh)	Left parsorbitalis (GMV)

4	Left parahippocampal (GMV)	Right superior frontal (CTh)	Right parahippocampal (MCu)

5	Left caudal middle frontal (MCu)	Left inferior parietal (CSA)	Left pars triangularis (CSA)

6	Right superior frontal (GMV)	Left parsopercularis (GMV)	Right precentral (MCu)

7	Right entorhinal (CTh)	Right lateral occipital (CTh)	Right rostral middle frontal (CSA)

8	Left lateral orbitofrontal (GMV)	Left inferior temporal (MCu)	Left isthmus cingulate (CSA)

9	Left superior frontal (GMV)	Right posterior cingulate (MCu)	Right lateral occipital (CTh)

10	Left pars triangularis (CSA)	Left postcentral (CSA)	Left entorhinal (CSA)

11	Right fusiform (CSA)	Left rostral middle frontal (MCu)	Right superior frontal (GMV)

12	Left middle temporal (GMV)	Left medial orbitofrontal (GMV)	Left parsopercularis (MCu)

13	Right caudal middle frontal (GMV)	Right supramarginal (MCu)	Left postcentral (CSA)

14	Left paracentral (CTh)	Right cuneus (CSA)	Left cuneus (CSA)

15	Right Cuneus (CTh)	Right parahippocampal (MCu)	Right lateral occipital (GMV)

### Features Contributing to Classification Performance

**Figure 6:**
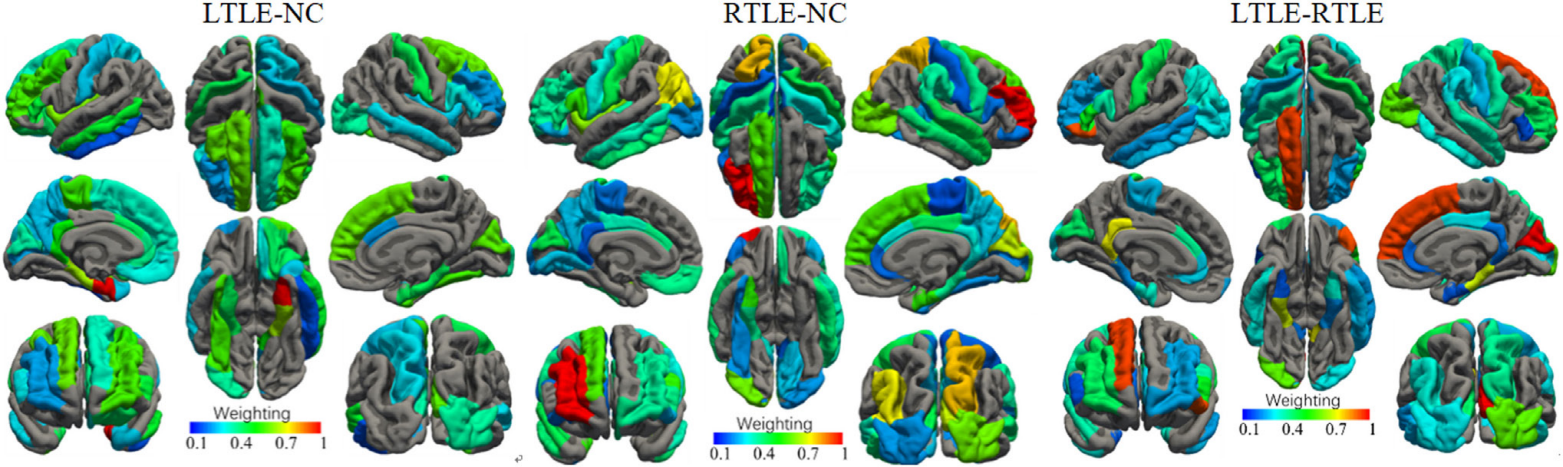
Weights of the cortical surface regions with prominent discriminative abilities determined by the support vector machine-recursive feature elimination. The brain regions with red color have high discriminative powers, while brain regions with blue color possess low discriminative abilities.

**Figure 7:**
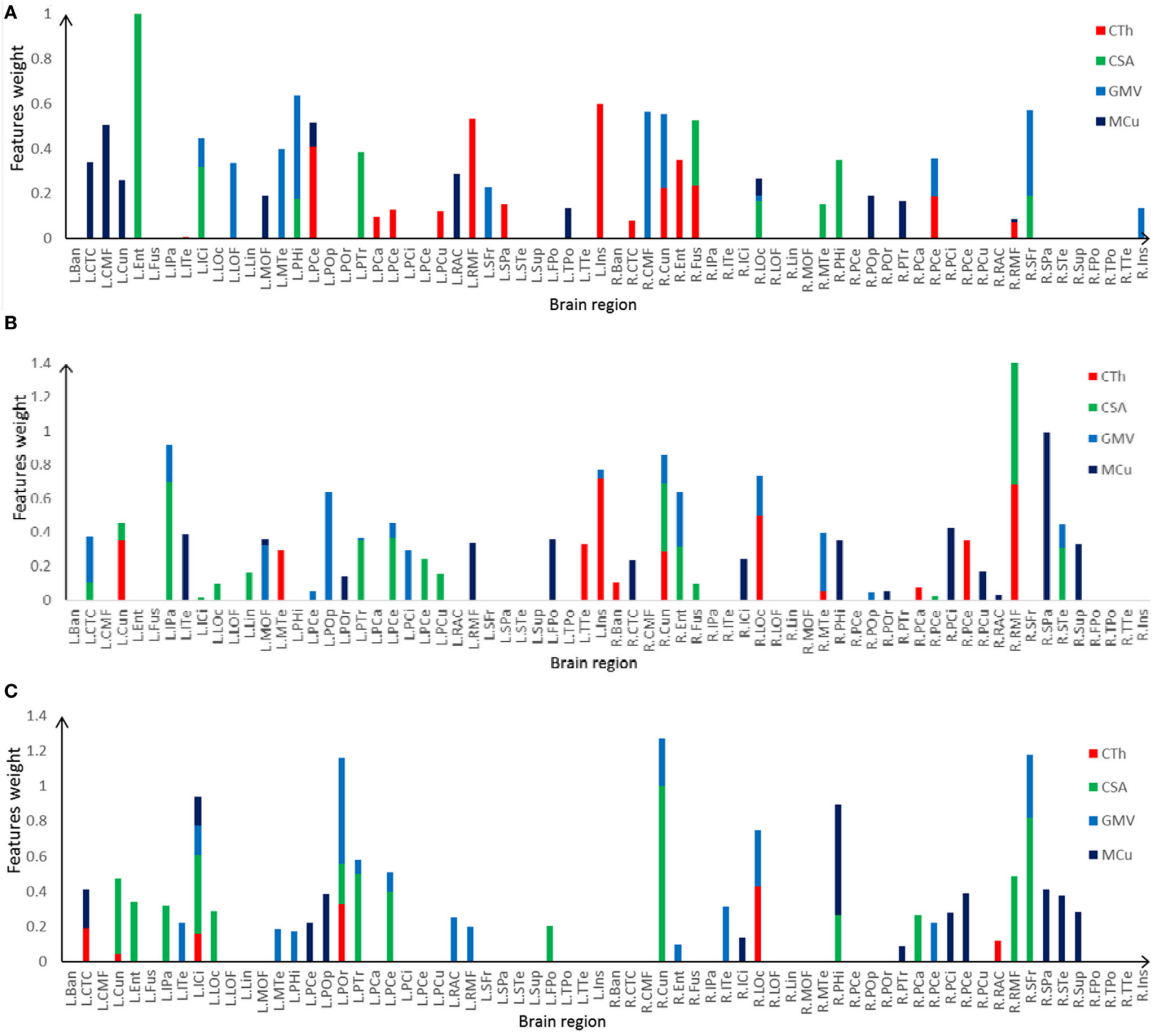
Selected features and their weights in left TLE (LTLE)-normal controls (NC), right TLE (RTLE)-NC, LTLE-RTLE classification by the support vector machine-recursive feature elimination using combined measures. **(A)** LTLE-NC, **(B)** RTLE-NC, **(C)** LTLE-RTLE. Cortical thickness (CTh), cortical surface area (CSA), GMV, and mean curvature (MCu) are denoted with red, green, dark green, and blue colors, and feature weights of different regions are accumulated according to the weights of four types of feature.

The most discriminative features determined by the SVM-RFE from the combined measures are illustrated in Table 3 and Figures 6 and 7. The top 15 features are listed in descending order by their weights (Table 3). The weights of features with prominent discriminative abilities were mapped into the brain template (Figure 6). To further illustrate the importance of all cortical surface features and compare their discriminative power, the weights of the features in classification are displayed in Figure 7.

## Discussion

Previous group-level statistical analysis of neuroimaging data has revealed some neuroanatomical and functional alterations between the TLE and healthy people (4, 7, 48). However, those group-level findings exhibited limited clinical application. Advanced techniques based on machine learning devised for pattern recognition could be applied for single subject prediction, which showed significant potential for assisting disease diagnosis (28, 32) and predicting treatment outcome (25, 49) of the patients with TLE in the individual level. In this study, by using cortical morphological features derived from brain MR images, the SVM classifier with efficient feature selection strategies could effectively differentiate the LTLE, RTLE, and NC. When combining the four different cortical measures, the classifier exhibited stronger discriminative power than the single measure.

Figure 2 illustrates the results of the cortical features analysis using QDEC with significance threshold set at *p* < 0.01. Patients with LTLE had significantly thicker thickness than NC in the left middle temporal, left insula, left parahippocampal cortex, right inferior temporal, and right cuneus, while the patients with RTLE showed significantly thicker thickness in the right precentral, right entorhinal cortex, left lateral occipital, and the left precuneus. Keller and Roberts (7) have summarized that morphological abnormalities widely occurred in the LTLE and RLTE, especially in the hippocampus, the parahippocampal gyrus, and the entorhinal cortex, which were mostly consistent with the current *t*-test findings. Functional MRI findings suggested that significant decreases of the ReHo were observed mainly in the DMN, including the precuneus, the posterior cingulate gyrus, the bilateral inferior lateral parietal, and the mesial prefrontal cortex cortex (8, 9).

With regard to the subcortical features, it could be seen from Table 2 that the ipsilateral hippocampus and thalamus volume were changed greatly in the LTLE and RTLE patients, which was consistent with the previous findings (10, 50). Hippocampal sclerosis is the most frequent histopathologic abnormality occurred in the patients with TLE (14). VBM studies have found widespread gray volume atrophy in the medial temporal cortex, including the hippocampus, thalamus, and amygdala (7). Our experimental results showed that ipsilateral hippocampus and thalamus volume variability could be a sensitive biomarker to detect the LTLE or RTLE patients, and it suggested that ipsilateral hippocampus and thalamus is vulnerable in the TLE patients. In addition to this, patients with TLE experience significant changes of volume in some regions including posterior cerebral cortex, central cerebral cortex, amygdala.

Among three investigated feature selection methods, the SVM-RFE had the most promising results, followed by the SCDRM and the *t*-test filtering approach. This was ascribed to its special scoring strategy for each feature in the training process of the SVM-RFE, which ranked all the features according to the scores assigned by the classifier and iteratively eliminated the features with the lowest scores (36, 41). It exhibited excellent performance in recent discriminative research (51, 52). The SCDRM performed effective classification with more than 80% accuracies for most comparisons, which demonstrated that the SCDRM could extract salient features with strong discriminative abilities. The SVM classifier achieved about 80% accuracy using the selected features. Arbabshirani et al. (53) systematically studied the relationship between significance level of group difference measured by two-sample *t*-test and the importance of the features contributing to classification. It was concluded that features that were statistically significant in the univariate analysis did not have strong discriminative ability and *vice versa*.

The most discriminative measures were the CSA and the GMV. The GMV was calculated by the product of CTh and CSA (54, 55). Reduction of the GMV was attributed to the decline of thickness (56), surface area (16), or both. Many studies only focused on voxel-based gray matter or white matter classification (27, 28). However, investigation on CTh and surface area could facilitate further understanding of the morphological changes in the cortex of patients with TLE. Comparing with other features such as clinical and neuropsychological data (57) and anatomical connectivity difference (27), the cortical features had much stronger power in discriminative analysis.

Regarding the spatial distribution of the dominant regions in combined features, the entorhinal CSA of the left hemisphere, the rostral middle frontal CSA of the right hemisphere, and the cuneus CSA of the right hemisphere were the most discriminative features in LTLE-NC, RTLE-NC, and LTLE-RTLE comparison, respectively. The left parahippocampal cortex, left rostral middle frontal, left insula, and right superior frontal also played a vital role in the LTLE discrimination, while the left inferior parietal, left insula, right cuneus, right lateral occipital, and right superior parietal had stronger power in the RTLE discrimination. These results provided some perspective value for the clinical diagnosis of the LTLE or RTLE, which were similar to the findings reported in previous studies (2, 16, 18, 56). The entorhinal cortex and parahippocampal cortex, which are located in temporal lobe, exhibited stronger discriminative performance in this study. Intracranial EEG analysis (58) suggested that abnormity in the entorhinal cortex may result in seizure generation. Another study revealed that parahippocampal was probably associated with patients with LTLE (59). Previous researches hypothesized that the left temporal lobe may be more vulnerable than the right side due to perinatal vascular, morphological changes, which were more diffuse and bilateral in the LTLE (26). As shown in Figure 6, the features contributing to the LTLE discrimination had a higher weighting score of 7.29 in the left brain hemisphere compared to the right hemisphere with 4.35, while the dominant features of the RTLE discrimination performed 8.52 in the right hemisphere compared to 7.18 in the left hemisphere. It illustrates that these cortical features correlated to the ipsilateral seizure onsets. The left insula and right cuneus showed predominant contribution to classification in this study, and they provided effective information for early diagnosis on both LTLE and RTLE. The widespread alterations in cortical features that extend beyond the hippocampus were associated with cognitive, intellectual, and executive function of the TLE (32).

The MRI visual inspection has been a conventional radiological procedure for the TLE diagnosis. Beyond some apparent abnormality in the TLE that could be identified through visual inspection by experienced physicians, several patients with epileptic discharge recording still showed no discernible MRI findings. This study demonstrated that the detection of brain cortical measures associated with TLE can be improved with computer-aided approach. There were 24.4% of the patients with LTLE and 23.5% of the patients with RTLE who did not show distinctive signs of these conditions in visual MRI inspection. More specifically, 10 of 41 LTLE and 8 of 34 RTLE were incorrectly identified in visual analysis. Our implemented method could reduce the incorrect cases into five LTLE and two RTLE patients, which demonstrated significant potential to improve diagnostic assessment, especially for patients who exhibited unremarkable lesions in standard radiological inspection.

This method has the potential to supplement visual assessment, especially for patients who do not have any obvious lesions in standard radiological examinations. The absence of visible lesion is one of the greatest challenges in epilepsy surgery, and correct patient lateralization (left vs. right seizure onset) and seizure localization play an important role for the treatment outcome.

This study has several limitations necessary to be mentioned. First, small sample size is a common pitfall existing in most of the similar studies. When the number of features is large in comparison to the sample size, the classifier tends to overfit the sample data. Therefore, more samples need to be collected in the further studies. Second, MRIs were obtained from two different scanners, which might need more specific strategy for feature extraction. However, previous research has proved that the FreeSurfer software had good test–retest reliability across scanner manufactures and field strengths (60). Although MR data acquired from different scanners certainly added extra variability that might have weakened the classifier to differentiate subjects, it also helped boost the method generalizability to the real-world situation. Finally, only the cortical surface features based on T1-MR data were investigated. Multimodal data such as fMRI, DTI, PET, and brain connectivity remained to be explored so as to provide either complementary or efficient information for accurate recognition, lateralization, and focus location in patients with TLE.

## Conclusion

In this study, three feature selection methods were investigated for detection of TLE using cortical surface features from brain structural MRI. The SVM-RFE strategy outperformed the other two methods (*t*-test and SCDRM) in discriminative analysis. The surface area and GMV had stronger discriminative ability than the CTh and MCu. Combined cortical surface features could improve the classification performance of the SVM classifier. Especially, the dominant regions with prominent discriminative power were mainly located in the temporal and in the frontal lobe, including the entorhinal cortex, rostral middle frontal, parahippocampal cortex, superior frontal, insula, and cuneus. The important brain features contributing to LTLE or RTLE discrimination mainly located in the ipsilateral hemisphere. It demonstrated that the cortical surface features could provide effective information in classification of left TLE, right TLE, and healthy subjects. The SVM-RFE could effectively extract the core features with strong discriminative ability and determine the salient brain regions associated with epilepsy.

## Ethics Statement

This study was carried out in accordance with the recommendations of Research Ethics Review Board of Guangdong 999 Brain Hospital with written informed consent from all subjects. All subjects gave written informed consent in accordance with the Declaration of Helsinki. The protocol was approved by the Guangdong 999 Brain Hospital.

## Author Contributions

CL contributed to the image processing, statistical analysis, and wrote the manuscript. SG contributed to the design and conceptualization of the study and revised the manuscript. LC and WW were involved in the MRI collection and interpretation of pathological mechanisms.

## Conflict of Interest Statement

The authors declare that the research was conducted in the absence of any commercial or financial relationships that could be construed as a potential conflict of interest.
